# Genome-Wide Analysis of the *Type-B Authentic Response Regulator* Gene Family in *Brassica napus*

**DOI:** 10.3390/genes13081449

**Published:** 2022-08-15

**Authors:** Jin-Jin Jiang, Na Li, Wu-Jun Chen, Yue Wang, Hao Rong, Tao Xie, You-Ping Wang

**Affiliations:** 1 Jiangsu Provincial Key Laboratory of Crop Genetics and Physiology, Yangzhou University, Yangzhou 225009, China; 2 Center for Plant Diversity and Systematics, Institute of Botany, Jiangsu Province and Chinese Academy of Sciences, Nanjing 210014, China; 3 School of Biological and Food Engineering, Suzhou University, Suzhou 234000, China; 4 Joint International Research Laboratory of Agriculture and Agri-Product Safety, The Ministry of Education of China, Yangzhou University, Yangzhou 225009, China

**Keywords:** *Brassica napus*, authentic response regulators, comparative studies, cytokinin, gene expression pattern

## Abstract

The type-B authentic response regulators (type-B ARRs) are positive regulators of cytokinin signaling and involved in plant growth and stress responses. In this study, we used bioinformatics, RNA-seq, and qPCR to study the phylogenetic and expression pattern of 35 type-B ARRs in *Brassica napus*. The BnARRs experienced gene expansion and loss during genome polyploidization and were classified into seven groups. Whole-genome duplication (WGD) and segmental duplication were the main forces driving *type-B ARR* expansion in *B. napus*. Several *BnARRs* with specific expression patterns during rapeseed development were identified, including *BnARR12/14/18/23/33*. Moreover, we found the *type-B BnARRs* were involved in rapeseed development and stress responses, through participating in cytokinin and ABA signaling pathways. This study revealed the origin, evolutionary history, and expression pattern of type-B ARRs in *B. napus* and will be helpful to the functional characterization of BnARRs.

## 1. Introduction

Cytokinin (CTK) is important in regulating plant growth and development as well as plant response to various stresses [1]. In *Arabidopsis*, the cytokinin signaling is phosphorylation-dependent, and is achieved through histidine kinases (AHKs), phosphotransfer proteins (AHPs), and authentic response regulators (ARRs) [2,3,4,5]. The *Arabidopsis* AHKs (AHK2/3/4) are transmembrane receptors for cytokinin that can be autophosphorylated to transfer the cytokinin signal to AHPs (AHP1~5) with functional redundancy [3,4,6,7,8,9]. Finally, phosphorylation signal was transferred from the AHPs to the aspartate residue in ARRs, which then regulate the transcription of target genes in the nucleus [10,11,12].

As reported in *Arabidopsis* and rice, the ARRs are generally divided into two subgroups, A- and B-type ARRs, which have also been reported as A-, B-, C-type, and *Arabidopsis* pseudoresponse regulators (APRR) in a few reports [13,14,15,16]. The type-A ARRs are known as negative regulators in cytokinin signaling, and they contain a short C-terminal extension besides the receiver domain [15]. Hitherto, type-A ARRs in *Arabidopsis* have been reported in controlling circadian period (ARR3/4), regulating meristem maintenance and regeneration (ARR4/7/8/15), root growth (ARR3), seed germination, and seedling growth (ARR4/5/6/7/15/16), as well as stomatal lineage ground cell division (ARR16/17) [17,18,19,20,21,22,23,24]. Unlike the A-ARRs, type-B ARRs in *Arabidopsis* contain a long C-terminal extension and a receiver domain in the N-terminal. They are positive regulators of phosphorelay signal transduction and transcriptional activators for A-ARRs [11,25]. The C-terminal extensions in B-ARRs are of diverse length, including a MYB-like binding domain (also named GARP domain) and a glutamine/proline-rich domain. The GARP domain is important in binding and activation of target genes, as well as interaction with other regulators [9,25,26,27]. Besides activating cytokinin signaling and cytokinin responsive genes, type-B ARRs (e.g., ARR1) may also act as negative-feedback regulators of cytokinin signaling [5,16,28,29,30]. The *Arabidopsis* B-ARRs are classified into three subfamilies. The members in subfamily 1 (ARR1/2/10/11/12/14/18) are broadly involved in cytokinin signaling [11,31,32], among which *ARR1*, *ARR10*, and *ARR12* are essential in regulating cytokinin signaling pathways [11,33]. The function of B-ARRs in subfamilies 2 (ARR13/21) and 3 (ARR19/20) remains to be characterized [12,34].

In *Arabidopsis*, the type-B ARRs are involved in plant growth and development, as well as stress responses [35,36,37]. Similar to other elements in the cytokinin signaling, the type-B ARRs are also functionally redundant [31,33,38]. Using the multiple mutants of *Arabidopsis*, the B-ARRs were characterized in regulating root and hypocotyl elongation, lateral root formation, shoot development, and callus induction [11,37,39,40]. In the quadruple mutant *arr1arr2arr10arr12*, nearly 78% ovules were arrested at the last stage of female gametophyte development, indicating they are necessary for megasporogenesis and megagametogenesis [41]. ARR1, ARR10, and ARR12 participated in shoot apical meristem (SAM) maintenance through directly activating *WUSCHEL* (*WUS*) expression [37]. Recently, Liu et al. (2020) showed that ARR1 inhibited shoot regeneration through competing with ARR12, a positive regulator of callus formation and shoot regeneration, to bind *CLAVATA3* (*CLV3*) promoter [42]. Comparative analysis of the ChIP-seq data confirmed that B-ARRs participated in various hormone responses and were important in hormone crosstalk [43]. In rice (*Oryza sativa*), mutation of type-B ARRs, ARR21/22/23, also resulted in defects in shoot, root, and flower growth, panicle architecture, and trichome formation [44], while *OsARR22* overexpression lines were cytokinin-hypersensitive, with significantly reduced root and flag leaf length, plant height, panical length, and lateral root number [45]. Cattani et al. (2020) found MdoARR1/8/10 regulated the transition from endo- to ecodormancy of apple bud via binding the *MdoDAM1* promoter [46].

Furthermore, type-B ARRs are also involved in plant response to biotic and abiotic stress processes [35]. The *Arabidopsis arr1arr10arr12* triple mutant was more resistant to aluminum (Al) stress than double mutants, while *ARR1* and *ARR12* overexpression plants were sensitive to Al, with inhibited root growth than the wild type, double, and triple mutants. This study confirmed the function of B-ARRs in Al-induced root development that is mediated by cytokinin and auxin [47]. ARR2 interacted with a salicylic acid (SA) response factor TGA2 and bound to the promoters of SA-responsive genes *PR1* and *PR2* to induce *Arabidopsis* resistance to *Pseudomonas syringae* in an SA-dependent way [48]. Besides *PR1* and *PR2*, the SA biosynthetic genes *SID1* and *SID2* were also induced in ARR2 overexpression plants inoculated with *P. syringae* [48]. Nguyen et al. (2016) showed that the *arr1arr10arr12* mutant was drought-tolerant with improved membrane integrity, anthocyanin biosynthesis, abscisic acid (ABA) sensitivity, and reduced stomatal size [49]. ARR1 is a positive regulator of freezing tolerance in *Arabidopsis*, through regulating A-ARRs’ expression. Additionally, the amino-terminal receiver domain of ARR1 was necessary for cold-responsive expression of *A-ARRs* (*ARR5/6/7/15*) [50]. It was also revealed that ARR2, 10, and 12 formed a complex to regulate photoperiod stress signaling in *Arabidopsis* [51].

Recently, genome-wide identification, expressional analysis, and functional prediction of type B-ARRs in rice, tobacco, tomato, and peach have been reported [52,53,54,55]. However, the ARRs in *Brassicas* have not been studied. Gene duplication is important to the genome expansion and plant evolution [56,57]. Oilseed rape (*Brassica napus* L., AACC, 2n = 38) is an important oil crop experienced whole-genome duplication (WGD) and triplication (WGT) during the evolution ~7500 years ago [58]. Its genomic complexity makes the gene functional analysis more challenging [59]. A detailed analysis of BnARRs would be helpful to know how they are involved in plant development and stress responses via cytokinin signaling and other putative signaling pathways. Here, we analyzed the evolution, structure, and expression profile of 35 type-B ARRs in *B. napus*. It is valuable for functional analysis of *BnARRs* in regulating rapeseed development and stress responses in the future.

## 2. Materials and Methods

### 2.1. Identification of Type-B ARRs in B. napus

The DNA and protein sequences were extracted from the *B. napus* genome database (http://www.genoscope.cns.fr/brassicanapus, accessed on 5 June 2022) [58]. Hidden Markov Model (HMM) profiles of the conserved response regulator (PF00072) and MYB_DNA-binding domain (PF00249) from Pfam (http://pfam.xfam.org/, accessed on 5 June 2022) were used to characterize the *BnARRs* with HMMER version 3.1 (https://www.ebi.ac.uk/Tools/hmmer/, accessed on 5 June 2022) [60,61]. ExPASy (https://web.expasy.org/protparam/, accessed on 5 June 2022) and IPC (http://isoelectric.org/index.html, accessed on 5 June 2022) were applied to acknowledge the protein length, molecular weight (Mw), and theoretical isoelectric point (pI) of BnARRs [62,63]. TargetP (version 2.0, http://www.cbs.dtu.dk/services/TargetP/, accessed on 5 June 2022) and Softberry (http://linux1.softberry.com/, accessed on 5 June 2022) were used to predict the subcellular localization of BnARRs [64].

### 2.2. Phylogenetic Analysis and Characterization of Type-B ARRs in B. napus

The BnARR proteins were aligned using clustalX (http://www.clustal.org/clustal2/, accessed on 7 June 2022) and edited by Jalview (http://www.jalview.org/, accessed on 7 June 2022). The sequence logos were visualized by WebLogo 3 (http://weblogo.threeplusone.com/, accessed on 7 June 2022). The *B-ARRs* of *B. rapa* and *B. oleracea* were obtained as mentioned above, from the database of *Brassicas* (https://www.brassica.info/, accessed on 7 June 2022). Eleven *Arabidopsis B-ARRs* were obtained from TAIR (https://www.arabidopsis.org/, accessed on 7 June 2022). The phylogenetic tree of B-ARRs in *A. thaliana*, *B. rapa*, *B. oleracea*, and *B. napus* was constructed with the neighbor-joining of MEGA 7.0 (https://www.megasoftware.net/, accessed on 7 June 2022), using the parameters as Xie et al. [65,66]. The gene structure of *BnARRs* were obtained from the genome data of *B. napus* (http://www.genoscope.cns.fr/brassicanapus, accessed on 5 June 2022) [58]. The conserved motif of BnARRs were identified by Multiple Em for Motif Elicitation (MEME, http://meme-suite.org/, accessed on 7 June 2022) [67]. The Amazing Optional Gene Viewer function of TBtools (https://github.com/CJ-Chen/TBtools, accessed on 7 June 2022) was used for visualization of gene feature and conserved motifs [68].

### 2.3. Chromosomal Location and Duplication Analysis of Type-B ARRs in B. napus

The chromosomal location of *type-B ARRs* were extracted from the genome annotation file of *B. napus*, and visualized using the MG2C (http://mg2c.iask.in/mg2c_v2.0/, accessed on 8 June 2022) [69]. The genome-wide protein sequence file of *A. thaliana* (https://www.arabidopsis.org/, accessed on 5 June 2022), *B. rapa* (http://brassicadb.org/brad/, accessed on 5 June 2022), *B. oleracea* (http://brassicadb.org/brad/, accessed on 5 June 2022), and *B. napus* were retrieved for alignment of homologs and orthologs. The synteny of B-ARRs among *B. napus* and other species were analyzed using Multiple Collinearity Scan toolkit (MCScanX, http://chibba.pgml.uga.edu/mcscan2/, accessed on 8 June 2022), and visualized by the Dual Systeny Plotter function in TBtools [70]. The gene duplication was analyzed with duplicat_gene_classifier program and MCScanX and classified into whole-genome duplication/segmental (collinear genes in collinear blocks), tandem (coherent repeat), proximal (nearby but not adjacent to chromosomal region), and dispersed (other type except for segmental, tandem, and proximal).

### 2.4. Analysis of Cis-Acting Elements in Promoters

The 2 kp upstream sequences of *BnARRs* were extracted by fadix command in SAMtools (version 1.4, http://samtools.sourceforge.net/, accessed on 8 June 2022) and analyzed in plant *cis*-acting regulatory DNA elements databases (PlantCARE, http://bioinformatics.psb.ugent.be/webtools/plantcare/html, accessed on 8 June 2022; New PLACE, https://www.dna.affrc.go.jp/PLACE/?action=newplace, accessed on 8 June 2022) [71,72,73]. Finally, the elements were visualized with the Simple BioSequence Viewer function in TBtools.

### 2.5. Gene Ontology (GO) Analysis

The GO annotation of *type-B ARRs* were obtained from the rapeseed genome database. The three GO categorization of BnARRs, molecular function (MF), biological process (BP), and cellular component (CC), were analyzed by Omicshare (http://www.omicshare.com/tools/, accessed on 9 June 2022) with a corrected *p* (FDR) < 0.05.

### 2.6. Plant Materials and Stress Treatments

To investigate the *BnARR* expression pattern throughout *B. napus* development, three replicates of root, cotyledon, hypocotyl, leaf, stem, shoot apical meristem (SAM), bud, flower, endosperm, silique at 14 days after pollination (DAP), seeds at five developmental stages (21, 28, 35, 42, 50 DAP) of *B. napus* line ‘J9712’ grown in field conditions were sampled for RNA-seq. The *BnARR* expression was normalized with log_10_FPKM values and plotted by Pretty Heatmaps in R (version 3.6.1, https://www.r-project.org/, accessed on 10 June 2022). For hormone treatment, five-week-old seedlings of *B. napus* line ‘J9712′, grown in a light incubator with 22 °C, 16 h light/8 h dark photocycle, were treated with 100 µM ABA, 100 µM kinetin (KT), 500 µM gibberellin (GA), 50 µM indoleacetic acid (IAA), and 10 µM strigolactone (SL). Three replicates of leaves were pooled respectively after 0 h, 1 h, 3 h, 6 h, and 12 h of treatment [74,75]. For osmotic, salt, and drought stresses, the ‘J9712′ seeds were germinated on 1/2 MS medium containing 150 mM mannitol, 150 mM NaCl, or 15% PEG. The 14-day-old seedlings were pooled after growing in a climate chamber under a photoperiod of 16 h light/8 h dark, 22 °C. For cold stress, the 12-day-old seedlings grown at 22 °C were transferred to 4 °C for 2 days. Three biological replicates of 10 seedlings were pooled for each treatment [76,77]. The *BnARR* expression under abiotic and hormone treatments were normalized with log_2_(FPKM ratio) compared with the control group.

### 2.7. Quantitative Real-time PCR (qPCR) Analysis

Total RNA from above samples were extracted with the RNAprep Pure Plant kit (TIANGEN BIOTECH, Beijing, China) and used for cDNA synthesis with HiScript^®^ II 1st Strand cDNA Synthesis Kit (+gDNA wiper) (Vazyme, Nanjing, China). qPCR primers of *BnARRs* listed in Table S1 were designed by Primer Premier 5.0 and synthesized by TSINKE Biotech (Beijing, China), *BnActin7* was the internal control. qPCR analysis was performed on a StepOnePlus^TM^ Real-time PCR system (Thermo, Waltham, MA, USA) using PowerUp SYBR Green Master Mixes (Thermo, USA). The *BnARR* expression level was calculated using 2^−ΔΔCt^ method [78].

### 2.8. Statistical Analysis

One-way ANOVA or *t*-test of SPSS version 19.0 (IBM, NewYork, NY, USA) was used for significant difference analysis of multiple samples or two samples at *p* < 0.05, respectively.

## 3. Results

### 3.1. Identification of Type-B ARRs in B. napus

In rapeseed, we identified 157 and 1046 proteins with the response regulator domain and MYB_DNA-binding domain, respectively. Only 45 proteins contained both response regulator and MYB binding domain, including ten orthologs of AtAPRRs that were excluded. Thus, a total of 35 *B. napus* proteins were taken as type-B ARRs for further analysis. Meanwhile, a total of 11, 15, and 18 type-B ARRs with both domains were identified in *A. thaliana*, *B. rapa* and *B. oleracea*. Interestingly, most type-B AtARRs were identified with orthologs in *B. napus*, except for AtARR13. The type-B ARRs (BnARR1~BnARR35) in rapeseed were named in serial numbers according to their chromosomal locations (Table S2). According to the triplication and duplication history of *B. napus*, we found ~47% B-ARRs were lost or rearranged during rapeseed evolution. Furthermore, we found BnARRs ranged from 187 (BnARR17) to 748 (BnARR18) amino acids (AA), the Mw ranged from 21.09 (BnARR17) to 83.04 (BnARR18) kDa, and the pI ranged from 4.84 (BnARR13) to 10.2 (BnARR2). Most BnARRs were acidic proteins since 80% of them were predicted with pI <7. All the BnARRs were predicted with nuclear localization (Table S2).

### 3.2. Sequence Alignment and Evolution Analysis of Type-B ARRs in B. napus

**Figure 1:**
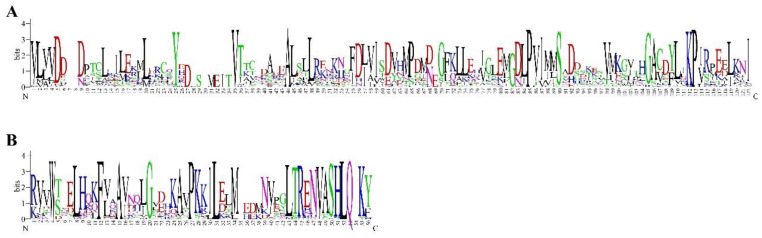
The conserved domains in type-B ARRs of *B. napus*. (**A**) The responsive regulator domain. (**B**) MYB_DNA-binding domain. Multiple sequence alignment of BnARRs were analyzed with Clustal X, and sequence logos were visualized by WebLogo 3.

Multi-protein sequence alignment confirmed that B-ARRs in rapeseed contained two main conserved domains (Figure 1). The 120 AA receiver domain (responsive regulator) in the N-terminal has a phosphorylated Asp residue in the center (Figure 1A). The DNA binding domain (~60 AA), also named B motif, was similar to the MYB_DNA-binding motif and is distinguished from other types of ARRs (Figure 1B).

**Figure 2:**
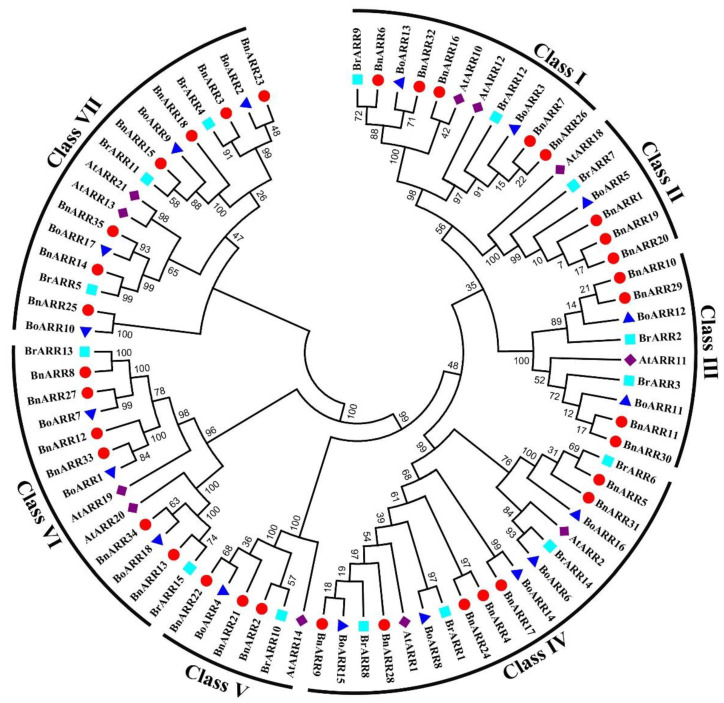
Phylogenetic tree of type-B ARRs in *B. napus*, *B. rapa*, *B. oleracea*, and *A. thaliana*. The numbers on branches represent the reliability percent of bootstrap values.

The phylogenetic tree of type-B ARRs in *B. napus*, *B. rapa*, *B. oleracea*, and *A. thaliana* classified them into seven groups (Class I~VII), each group contained 5, 3, 4, 7, 3, 6, and 7 BnARRs, respectively (Figure 2). In Class IV, 16 ARRs from *Brassicas* were orthologs of AtARR1 and AtARR2. The nine ARRs clustered in Class I were orthologs of AtARR10 and AtARR12. These BnARRs might play important roles in cytokinin response processes since AtARR1/2/10/12 have been reported with multiple functions in cytokinin signaling and plant development [3,4,32,35,37,79,80].

### 3.3. Gene Structure and Conserved Motifs of Type-B ARRs in B. napus

**Figure 3:**
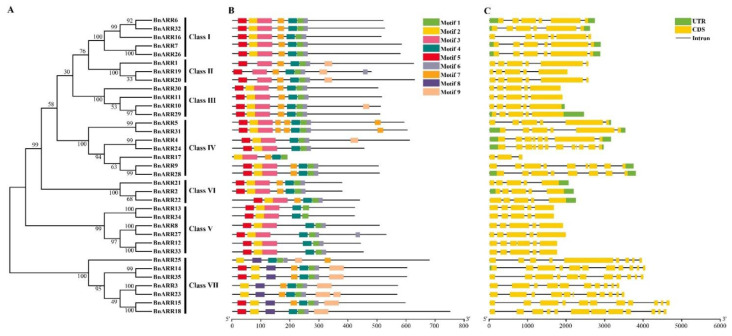
Gene structure and motif composition of *type-B ARRs* in *B. napus*. (**A**) Phylogenetic tree of BnARRs. (**B**) Conserved motifs in BnARRs. Motifs 1~10 are displayed with different colored boxes. The detailed motif structure is showed in Figure S1. (**C**) Gene structure of *BnARRs*. UTR, untranslated region; CDS, coding sequence.

Gene structure is correlated with expression and function divergence, the coding regions responsible for various gene functions may be due to the alterations in exon-intron structure and/or amino acid substitutions [81,82]. We found the intron number of *BnARRs* ranged from 2 to 11, of which 23 *BnARRs* contained four or five introns. The exon number ranged from 3 to 12 (Figure 3). Furthermore, the exon number in Class IV and VII was 3~12 and 9~12, respectively. The exon length and number was more consistent in Class I, II, III, VI, and V. In general, *BnARRs* in the same phylogenetic branch showed similar structures, but the intron length varied in some groups, such as *BnARR4*/*BnARR24*, which may contribute to the functional differentiation of duplicated *ARRs*.

Nine conserved motifs of BnARRs were identified, ranging from 15 to 50 AA (Figure 3 and Figure S1). Motifs 2, 3, and 5 were located in the receiver domain, and motifs 1 and 4 were located in the DNA-binding domain. In addition, motif 6 was presented in 32 BnARRs (80%) in seven groups, and motif 7 was existed in 26 BnARRs. Motif 9 was mainly identified in Class II and VII, and also found in two members of Class III and IV. Motif 8 was specific to Class VII. The specific motif patterns may also lead to functional divergence of BnARRs in different groups.

### 3.4. Chromosomal Location and Synteny of BnARRs

**Figure 4:**
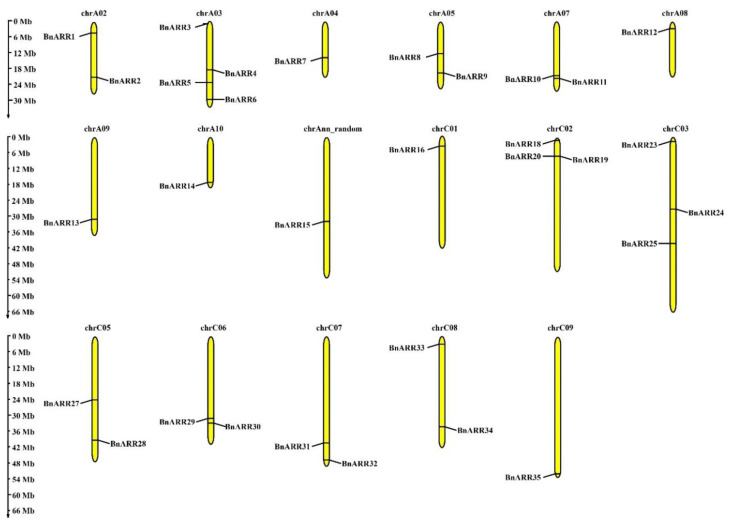
The distribution of *type-B ARRs* on rapeseed chromosomes. Mb, megabase.

A total of 31 *BnARRs* (19 genes on A subgenome and 16 on C subgenome) were physically localized on rapeseed chromosomes, except for four members located on Ann_random and Cnn_random due to the incomplete *B. napus* genome (Figure 4). We found all the *BnARRs* with two to seven homologs on A and C subgenomes, such as *BnARR2*/*21*/*22* and *BnARR10*/*11*/*29*/*30*.

**Figure 5:**
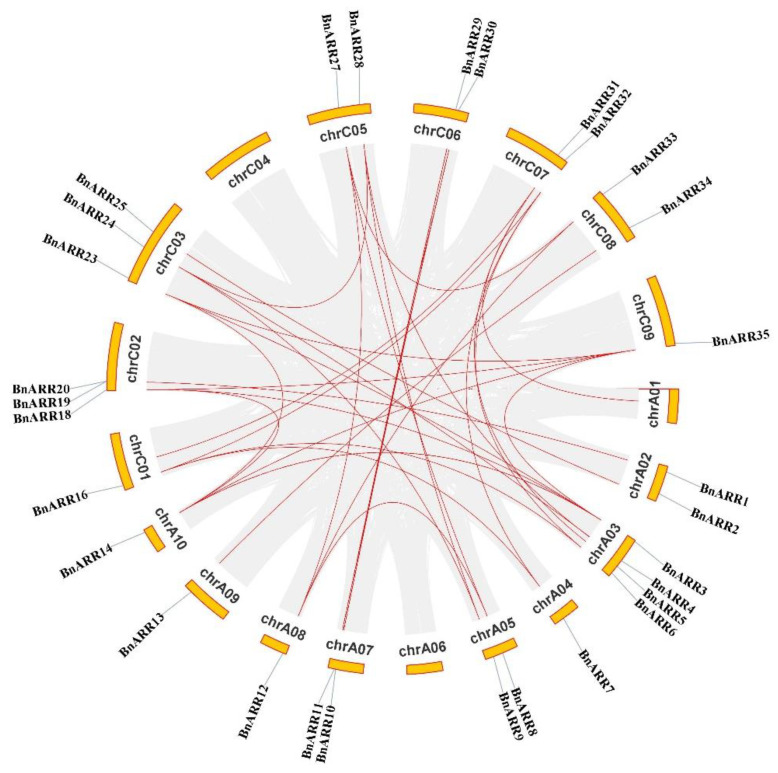
Synteny of *type-B ARRs* in *B. napus*. The gray lines represent synteny blocks in rapeseed genome, and red lines represent *BnARR* gene pairs.

Duplication is important to plant evolution and adaptation [83]. Based on the synteny analysis, we found the *BnARRs* have experienced different types of duplication events. Twenty-eight *BnARRs* (80%) were derived from WGD/segmental events, only one *BnARR* derived from a tandem event, two *BnARRs* derived from proximal events, and four genes resulted from dispersed events (Figure 5, Table S3). In addition, 26 paralogous gene pairs were identified, indicating gene duplication, especially WGD/segmental events, was the main force driving *type-B ARR* expansion in *B. napus*.

**Figure 6:**
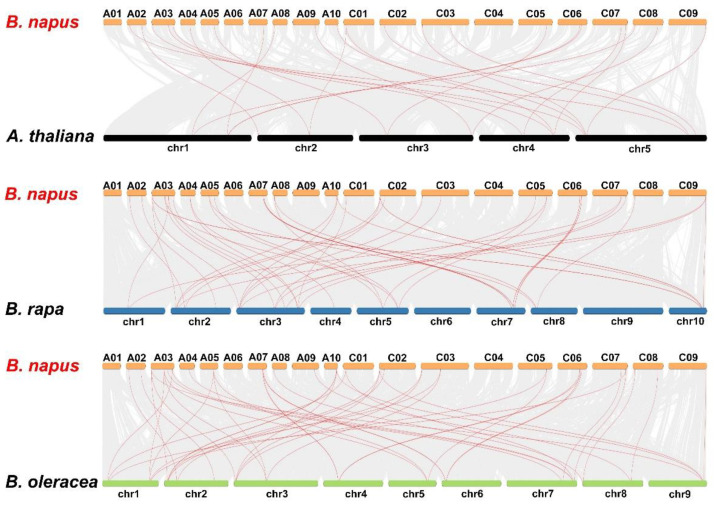
The collinearity of *type-B ARRs* in *B. napus* and three ancestral species. Gray lines represent collinear blocks among *B. napus*, *B. rapa*, *B. oleracea*, and *A. thaliana*; red lines indicate the syntenic *type-B ARR* pairs.

The expansion and evolution of *ARRs* in Brassicaceae was revealed by synteny analysis among *B. napus*, *A. thaliana*, *B. rapa*, and *B. oleracea* (Figure 6, Table S4). About 82.9% (29/35) of *BnARRs* had syntenic relationship to *ARRs* in other species. Specifically, 27, 27, and 22 *BnARRs* were predicted with synteny to *B. rapa*, *B. oleracea*, and *A. thaliana*, respectively. We found 29 *BnARRs* were inherited from *B. rapa* or *B. oleracea*, while the remaining six *BnARRs* were novel members after genome duplication.

Based on the *Ka* (non-synonymous substitutions per site), *Ks* (synonymous substitutions per site), and *Ka/Ks* ratio, we predicted the selective pressure of *ARR* gene pairs in *B. napus*, *B. rapa*, *B. oleracea*, and *A. thaliana* (Table S4, Figure S2). The *Ka/Ks* ratio of gene pairs in *B. napus*–*B. napus*, *B. napus*–*B. rapa*, *B. napus*–*B. oleracea*, and *B. napus*–*A. thaliana* were 0.4476, 0.4588, 0.4465, and 0.3370, respectively. As reported, *Ka/Ks* ratio >1, =1, and <1 represented positive selection, neutral mutation, and purifying selection, respectively [84]. This indicated that most *BnARR* pairs experienced strong purifying selection. Furthermore, the *type-B ARR* gene pairs between *B. napus*–*B. napus*, *B. napus*–*B. rapa*, *B. napus*–*B. oleracea*, and *B. napus*–*A. thaliana* were diverged 0.1596, 0.0979, 0.1031, and 0.2671 million years ago (Mya), respectively. Thus, the *ARRs* in *B. napus*–*A. thaliana* were diverged earlier than in other comparisons.

### 3.5. GO Enrichment and Expression Profiles of Type-B ARRs in B. napus

**Figure 7:**
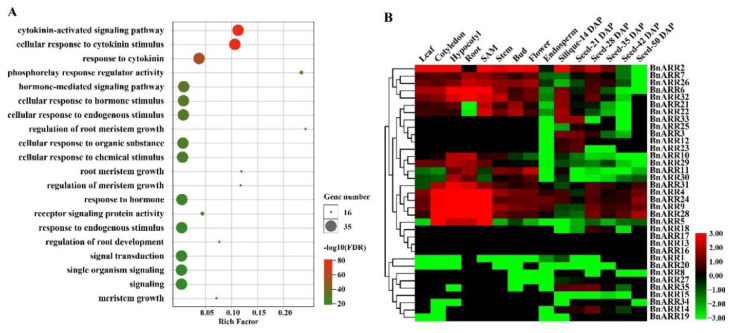
GO enrichment and spatiotemporal expression pattern of *type-B ARRs* in *B. napus*. (**A**) The top 20 GO terms enriched by *BnARRs*. Rich factor means the ratio of *BnARR* gene number to transcript number in each term. (**B**) Heatmap of *BnARRs* expression with log_10_ FPKM. DAP, days after pollination; SAM, shoot apical meristem.

To acknowledge the putative function of *type-B ARRs* in *B. napus*, we enriched these genes with the GO terms, and the top 20 enriched terms included biological process of cytokinin-activated signaling pathway (GO: 0009736), cellular response to cytokinin stimulus (GO: 0071368), and response to cytokinin (GO: 0009735). This indicated that type-B ARRs participated in the cytokinin signaling process of *B. napus*. Moreover, ~42.22% of *BnARRs* were enriched in the molecular function of phosphorelay response regulator activity (GO: 0000156), agreeing with the function of the receiver domain in BnARRs (Figure 7A, Table S5).

Based on the RNA-seq data of different tissues and organs representing *B. napus* development, we found ~74.29% of the *type-B ARRs* were expressed in one or more tissues/organs with FPKM value > 1, while the rest *BnARRs* (e.g., *BnARR13*, *BnARR16*, and *BnARR17*) were non-expressed genes (Figure 7B, Table S6). *BnARR2*, *BnARR4*, *BnARR9*, and *BnARR24* were expressed in more than 12 tissues/organs. Eight *BnARRs* were expressed in three or fewer tissues/organs, such as *BnARR14/18* expressed in 28 DAP seed, *BnARR12/23/33* expressed in 14 DAP silique and 21 DAP seed. The different *BnARR* expression pattern might be related to their functional diversification, and the *ARRs* with high expression level in specific tissue/organ may take part in the processes of plant development.

**Figure 8:**
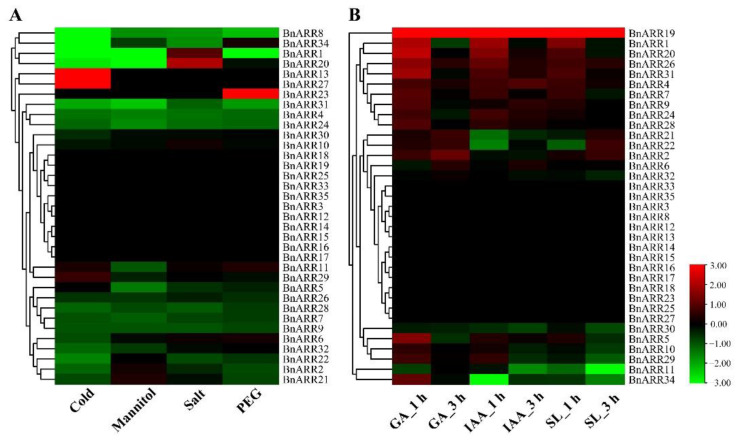
*Type-B BnARR* expression in response to abiotic and hormone treatments. (**A**) *BnARR* expression under abiotic stresses. (**B**) *BnARR* expression under hormone treatments.

We analyzed the *BnARR* expression under abiotic stresses and hormone treatments, but not all the members could respond to these treatments. Most *BnARRs* with response to abiotic stresses were down-regulated under cold, mannitol, salt, and PEG treatments, such as *BnARR1/4/7/8/9/20/24/28/31/34*. However, *BnARR20* was up-regulated by salt stress, *BnARR13* and 27 were up-regulated by cold stress, and *BnARR23* was up-regulated by drought stress (Figure 8A). Furthermore, the members in class III were down-regulated under IAA and SL treatments, such as *BnARR10/11/29/30*. *BnARR2/21/22* in class VI were up-regulated under GA treatment. *BnARR7/26* in class I, *BnARR1/19/20* in class II, and *BnARR4/9/24/28/31* in class IV were up-regulated under GA, IAA, and SL treatments (Figure 8B).

### 3.6. Cis-Acting Elements in Type-B BnARR Promoters

**Figure 9:**
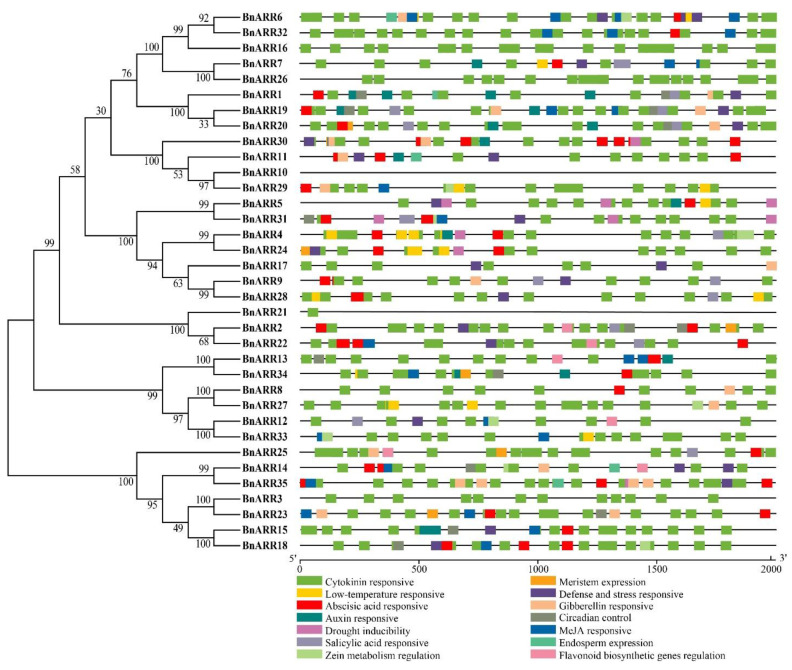
*Cis*-acting elements in the *type-B BnARR* promoters.

Gene promoters contain a large number of *cis*-acting elements that can specifically bind to proteins involved in the initiation and regulation of gene transcription [85]. Here, we analyzed the *type-B BnARR* promoters, and classified the *cis*-acting elements into four types, abiotic responsiveness, hormones responsiveness, plant growth and development, and other basic promoter elements like TATA-box (Figure 9, Table S7). The circadian control (circadian motif), zein metabolism regulation (O2-site motif), meristem expression (CAT-box), endosperm expression (GCN4 motif), phytochrome down-regulation expression, seed-specific regulation (RY element), endosperm-specific negative expression (AACA motif), root-specific elements (motif I), differentiation of the palisade mesophyll cells (HD-Zip 1 motif), and light-responsive elements (TCT motif, G-box, GT1 motif, and AE-box) were related to plant growth and development. Furthermore, the light-responsive elements were ubiquitous in the *BnARR* promoters, indicating that *BnARRs* may be involved in the light-regulated plant development. As to the hormone-responsive elements, we found the cytokinin and abscisic acid responsive elements were enriched in *BnARR* promoters. The existence of anaerobic induction (ARE motif), defense and stress responsiveness (TC-rich motif), low-temperature responsiveness (LTR motif), MYB binding site involved in drought-inducibility (MBS motif), anoxic-specific inducibility (GC motif), and wound responsiveness (WUN motif) indicated that *BnARRs* may also regulate plant response to abiotic stresses.

### 3.7. The Expression Pattern of Type-B BnARRs under Cytokinin and ABA Treatments

**Figure 10:**
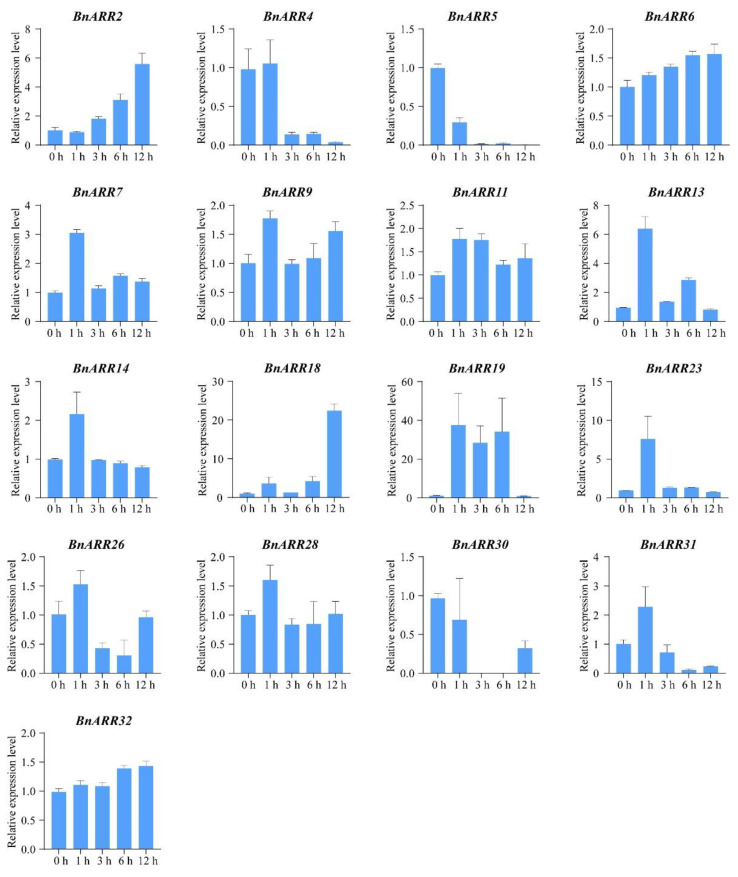
*Type-B BnARRs* expression in response to CTK treatment. The data was represented as mean ± standard deviation (*n* = 3).

**Figure 11:**
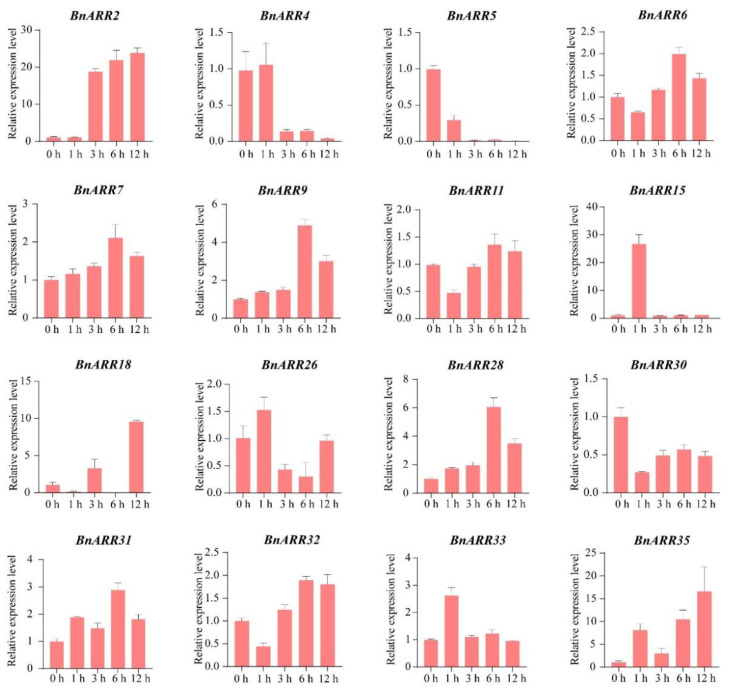
*Type-B BnARRs* expression in response to ABA treatment. The data was represented as mean ± standard deviation (*n* = 3).

As mentioned above, the *cis*-acting elements associated with ABA and CTK responses were enriched in *BnARR* promoters. Thus, we used qPCR to analyze the *BnARR* expression pattern under different time of ABA and CTK treatments. The *BnARR4/5/30* were down-regulated under CTK treatment, while *BnARR18/19/23* were strongly up-regulated. *BnARR18* and *BnARR23* were obviously up-regulated after 12 h and 1 h of CTK treatment, respectively. *BnARR19* was consistently up-regulated during 1~6 h of CTK treatment. In addition, 12 *BnARRs* were slightly induced or repressed by CTK (Figure 10). Under ABA treatment, *BnARR2*/*9*/*15/28/35* were up-regulated, but with the highest expression level at different hours after ABA treatment. Moreover, ABA repressed the expression level of *BnARR4* and *BnARR5* with the minimum expression at 12 h of ABA treatment. We found eight *BnARRs* were slightly up-regulated (e.g., *BnARR6* and *BnARR7*) or down-regulated (e.g., *BnARR30*) after ABA treatment (Figure 11). These results indicated the *BnARRs* might be involved in CTK and ABA signaling pathways.

## 4. Discussion

Cytokinin is important in regulating plant development and response to biotic and abiotic stresses [1]. Among the two main types of ARRs (A- and B-ARRs) involved in cytokinin signaling [37], type-B ARRs are transcription factors that can be activated by phosphorylation of the Asp residue in the receiver domain [1]. Hitherto, the B-ARRs have been studied in *Arabidopsis*, rice, tomato, tobacco, and peach, but not in oil crop *B. napus* [11,52,53,54,55]. In this study, 35 B-ARRs were identified in rapeseed based on the two conserved domains. The gene structure, chromosomal location, duplication event, *cis*-acting element, and expression patterns of these BnARRs were analyzed. The type-B BnARRs were divided into seven classes, which were consistent with that in *Arabidopsis* [86]. The gene structure and conserved motifs of *BnARRs* in the same class were similar but differed among different groups. The different intron-exon structure of *BnARRs* might be due to chromosome rearrangement and translocation during polyploidization. Recently, introns have been proved with important functions in regulating gene expression [87,88]. We found the intron number varied a lot among *BnARRs*, which might be valuable to *BnARR* evolution.

*B. napus* is a crop derived from natural hybridization of diploid parents, *B. rapa* and *B. oleracea*. It has undergone 72× genome multiplication compared with the basal angiosperm *Amborella trichopoda* [58]. The 35 *BnARRs*, 15 *BrARRs*, 18 *BoARRs*, and 11 *AtARRs* were identified with collinearity among different species. WGD/segmental and tandem play prominent roles in the evolution of many eukaryotic species [89,90]. In *B. napus*, 80% *B*-*ARRs* (28/35) experienced WGD/segmental duplication, 11.43% *BnARRs* (4/35) resulted from dispersed duplication, only one *BnARR* and two *BnARRs* were originated from tandem and proximal duplication, respectively. Other gene families in rapeseed have also experienced a WGD/segmental duplication event, including 73.81% *BnLBDs*, 92.3% *BnKCSs*, 70.98% *BnNPFs*, 96.96% *BnATGs*, and 73.4% *BnMATEs*. Thus, WGD/segmental duplication is a main force for gene family expansion in rapeseed [66,91,92,93,94]. However, not all the duplicated genes were retained; some genes were quickly erased to maintain the genome stability [95]. The genome and synteny analysis confirmed that *B. rapa* and *B. oleracea* endured WGT 20 to 40 Mya [58,96]. Theoretically, about 47% of B-ARRs were lost during rapeseed evolution, and this might be due to the purifying selection. Furthermore, the *AtARR13* ortholog was not found in rapeseed, which might be redundant during rapeseed genome evolution.

Based on the expression profile of *B-ARRs* during rapeseed development, we found three putative pseudogenes (BnARR13/16/17) that were not expressed in all analyzed tissues/organs. Ten *BnARRs* were expressed throughout rapeseed development and might be important to rapeseed growth. The different expression pattern of *BnARRs* might be caused by the neo-, sub-, and non-functionalization after gene duplication. Moreover, we identified a few BnARRs (e.g., *BnARR4/7/8/9/24/28/31*) that were down-regulated under cold, mannitol, salt, and PEG stresses, while *BnARR13/20/23/27* were up-regulated by salt, cold, or drought stress. Furthermore, the *BnARRs* in class III were down-regulated by IAA and SL, the members of class VI were up-regulated by GA. A few members in class I (*BnARR7/26*), class II (*BnARR1/19/20*), and class IV (*BnARR4/9/24/28/31*) were up-regulated by GA, IAA, and SL. *Cis*-acting elements are important in regulating gene expression [85]. The abiotic responsive elements, hormone-responsive elements, plant growth-related elements, and development-related elements were enriched in the *type-B BnARR* promoters. We confirmed that 17 and 16 *BnARRs* were induced or repressed by CTK and ABA, respectively. These genes may participate in hormone-regulated plant development and stress responses, since ABA and CTK are important endogenous messengers in plants and play vital roles in regulating plant development and adaptation [1,97]. However, only six *BnARRs* were significantly up- or down-regulated by cytokinin. In *Arabidopsis*, *A-ARR* expression was more induced by cytokinin than the *B-ARRs* [49]. GO analysis also enriched the *type-B BnARRs* in CTK-related terms, such as CTK-activated signaling pathway, cellular response to CTK stimulus, and response to CTK. As reported, B-ARRs were activated through phosphorylation in the receiver domain after CTK treatment and bound to the target genes in a CTK-dependent manner [35]. *AtARR1/10/12* were negative regulators in plant response to drought stress; the triple mutants were drought-tolerant compared with the wild type [49]. In plants, ABA regulates numerous biological processes, including seed dormancy and germination, lateral root formation, and stress responses. It could broadly regulate the expression of stress-responsive genes [98]. In this study, the *BnARRs* (e.g., *BnARR2*/*9*/*15/28/35*) with significant expressional changes under ABA treatment may also be involved in plant response to abiotic stresses.

## 5. Conclusions

We comprehensively analyzed the 35 type-B ARRs in *B. napus*. This gene family experienced expansion and loss during rapeseed polyploidization, and these BnARRs were grouped into seven classes. The GO enrichment, temporospatial expression pattern, and response to abiotic and hormone treatments suggest that *type-B BnARRs* played important roles in rapeseed growth, development, and stress responses, especially via ABA and cytokinin signaling pathways. In general, these findings will be helpful to the further functional investigation of type-B BnARRs.

## Data Availability

Not applicable.

## Supplementary Materials

The following supporting information can be downloaded at: https://www.mdpi.com/article/10.3390/genes13081449/s1, Figure S1: Amino acid sequence logos of conserved domains in type-B ARRs of *B. napus*; Figure S2: Box-plot of Ka, Ks, Ka/Ks value and divergence time of *type-B ARR* gene pairs. Mya, million years ago; Table S1: The qPCR primers used in this study; Table S2: The information of type-B ARR family members in *B. napus*; Table S3: Duplication type of *type-B ARRs* in *B. napus*; Table S4: Ka/Ks calculation of the duplicated *type-B ARR* gene pairs. (A) *B. napus*–*B. napus*. (B) *B. napus*–*B. rapa*. (C) *B. napus*–*B. oleracea*. (D) *B. napus*–*A. thaliana*; Table S5: GO enrichment analysis of *type-B ARRs* in *B. napus*; Table S6: The RNA-seq data (FPKM) of *type-B BnARRs* in different tissues and developmental stages; Table S7: *Cis*-acting element analysis of *type-B BnARRs*.
