# Supplementary figures and images for: Genome-Wide Analysis of the Type-B Authentic Response Regulator Gene Family in Brassica napus
Source: Genes (Basel). 2022 Aug 15;13(8):1449. doi: 10.3390/genes13081449 (PMC9408017; doi:10.3390/genes13081449)

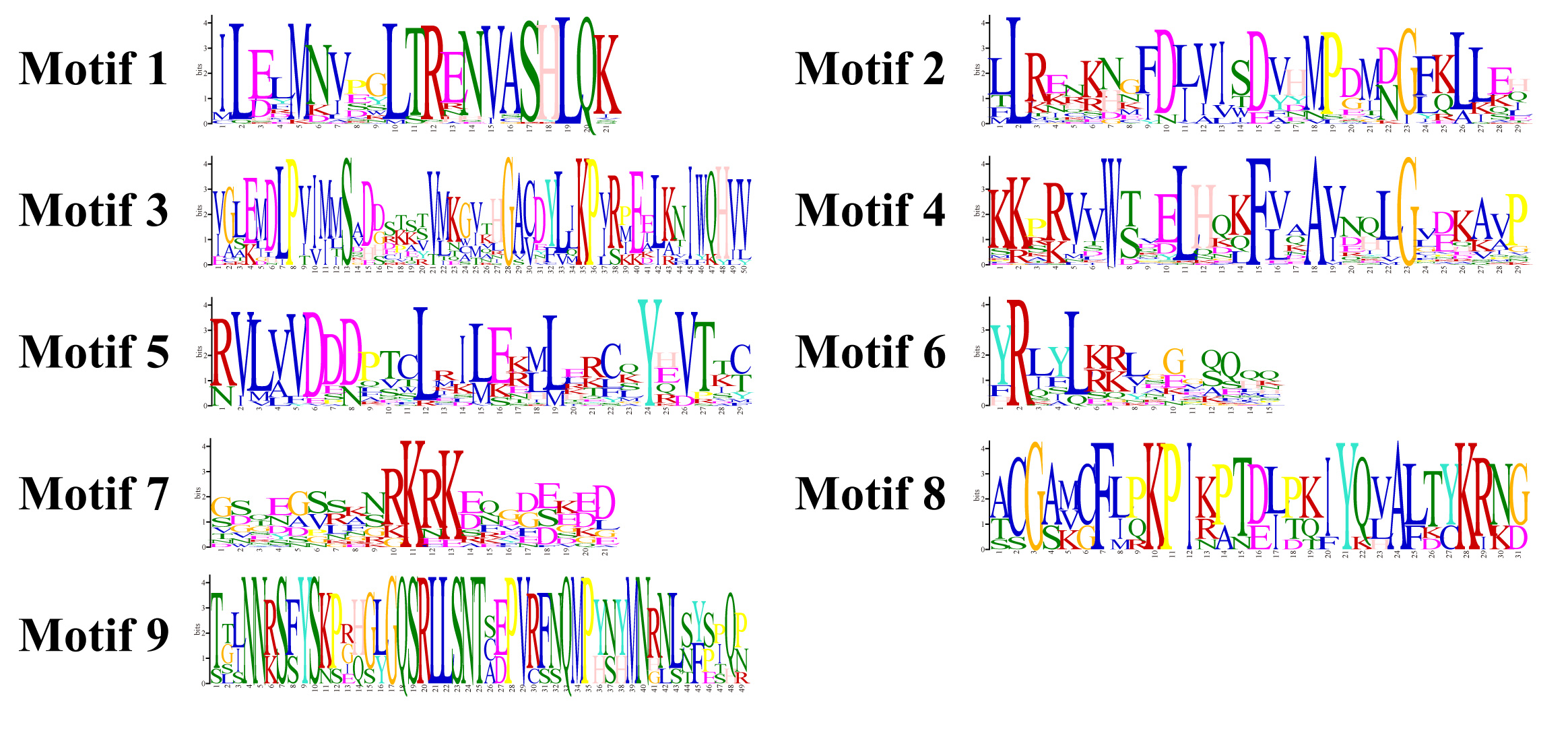

Supplement: Supplementary file 1 [file genes-13-01449-s001.zip › Figure S1.jpg]

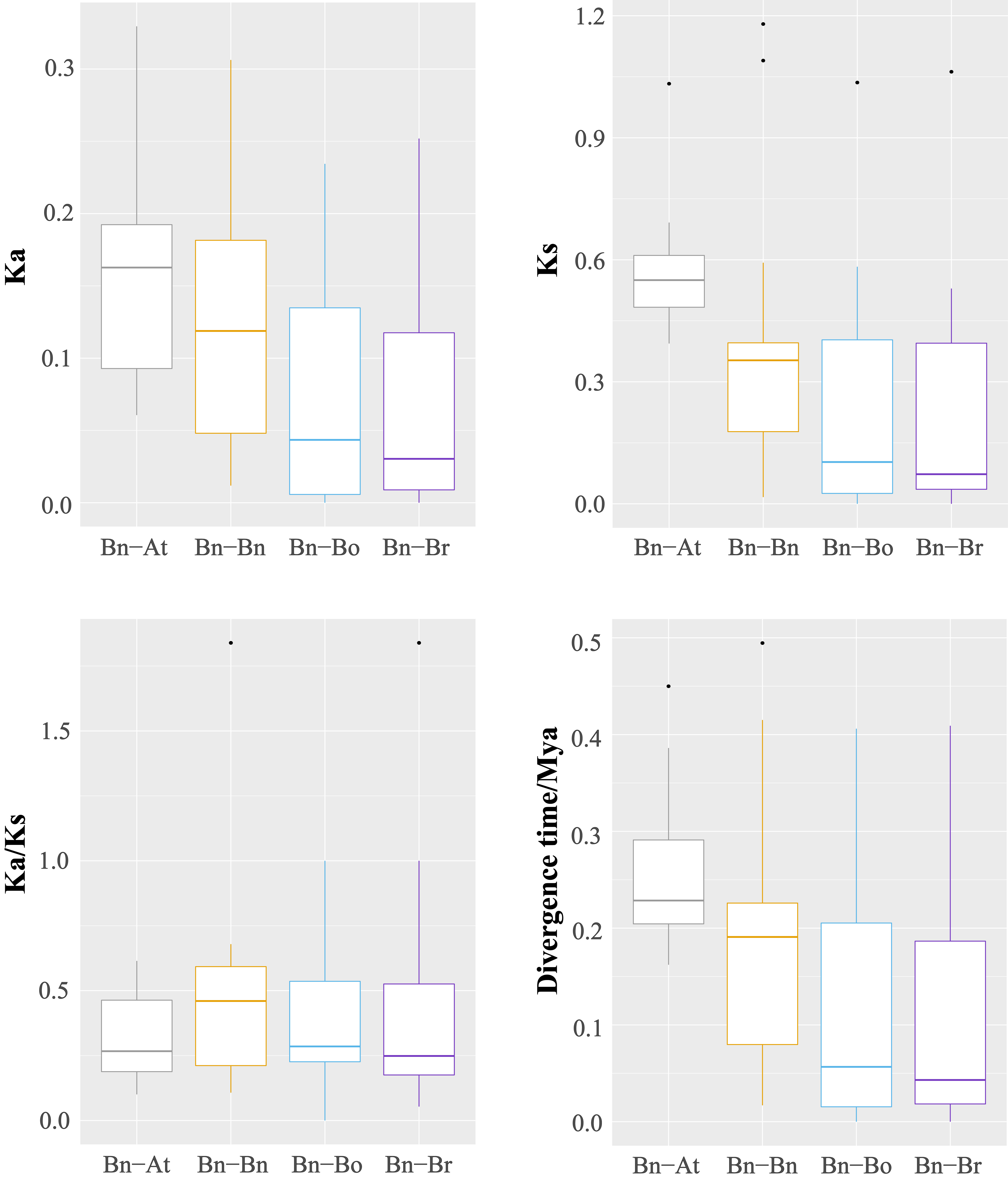

Supplement: Supplementary file 1 [file genes-13-01449-s001.zip › Figure S2.jpg]
